# Effect of Inflammation on the Process of Stroke Rehabilitation and Poststroke Depression

**DOI:** 10.3389/fpsyt.2019.00184

**Published:** 2019-04-11

**Authors:** Meidan Fang, Lili Zhong, Xin Jin, Ranji Cui, Wei Yang, Shuohui Gao, Jing Lv, Bingjin Li, Tongjun Liu

**Affiliations:** ^1^Department of General Surgery, Second Hospital of Jilin University, Changchun, China; ^2^Jilin Provincial Key Laboratory on Molecular and Chemical Genetic, Second Hospital of Jilin University, Changchun, China; ^3^Department of Oncology and Hematology, Second Hospital of Jilin University, Changchun, China; ^4^Department of Gastrointestinal Colorectal Surgery, China-Japan Union Hospital of Jilin University, Changchun, China; ^5^Chang Chun University of Chinese Medicine, Changchun, China

**Keywords:** stroke, immune/inflammation, rehabilitation, poststroke depression, pharmacotherapy

## Abstract

A considerable body of evidence has shown that inflammation plays an important role in the process of stroke rehabilitation and development of poststroke depression (PSD). However, the specific molecular and cellular mechanisms involved remain unclear. In this review, we summarize how neuroinflammation affects stroke rehabilitation and PSD. We mainly focus on the immune/inflammatory response, involving astrocytes, microglia, monocyte-derived macrophages, cytokines (tumor necrosis factor alpha, interleukin 1), and microRNAs (microRNA-124, microRNA 133b). This review provides new insights into the effect of inflammation on the process of stroke rehabilitation and PSD and potentially offer new therapeutic targets of stroke and PSD.

## Introduction

Stroke is defined as permanent tissue damage caused by a sudden loss of brain blood supply as a result of occlusion or a hemorrhage. Stroke includes two main types, ischemic stroke and intracerebral hemorrhage (ICH). Approximately 85% of strokes belong to the ischemic type and 12% are ICHs (3% are subarachnoid hemorrhage). Neural plasticity can be affected by different risk factors of stroke, medical management, and anti-inflammatory interventions during the process of stroke rehabilitation.

The immune/inflammatory response can be triggered by several factors, such as ischemia or hemorrhage. Microglia, astrocytes, and endotheliocytes are involved in immune/inflammatory activation induced by ischemic stroke. These cells can communicate with each other by proinflammatory and anti-inflammatory factors, such as cytokines and adhesion molecules. Inflammatory cells, such as neutrophils and macrophages, are activated, reach the ischemic area, and contribute to the inflammatory response (1). These immune responses following the initial ischemic insult can be long lasting and subsequently modulate synaptic plasticity alterations during the process of stroke rehabilitation (2). In ICH, ambient microglia and astrocytes can also exert modulatory effects during ICH rehabilitation (3).

Poststroke depression (PSD), a critical psychiatric complication of stroke, involves several major symptoms including sleep and appetite disturbance, psychomotor agitation, and fatigue (4). As the inflammatory response may modulate neuroplasticity during stroke and altered neuroplasticity may be associated with the onset of PSD, the stroke-induced immune response in the brain can also affect the PSD process. It was found that several inflammatory markers, pro-inflammatory cytokines, and the pro-inflammatory/anti-inflammatory ratios were increased and the complementary expression was reduced in the PSD process (5).

In this review, we describe the two different types of stroke (ischemic stroke and ICH) and respectively summarize the effects of inflammation on the process of stroke rehabilitation. We then describe PSD and summarize the effects of inflammation on PSD. Finally, we discuss the potential efficacy of using anti-inflammatory medication for stroke rehabilitation and PSD prevention.

## Stroke

Stroke refers to several conditions caused by occlusion or a hemorrhage of the brain blood vessels. Stroke is a worldwide neurological disease with few effective treatments and preventative measures (6). Neuroinflammation involves damage-associated molecular patterns, instead of microbial pathogens (7). Importantly, neuroinflammation plays a key role in several neurological diseases such as a hemorrhage and ischemia (8). There are certain complicated connections between immune/inflammatory processes and stroke rehabilitation.

### Ischemic Stroke

Ischemic stroke, the most common type of brain ischemic injury in humans, is the leading cause of mortality and long-term disability (9). Ischemic stroke is mainly caused by an ischemic core induced by brain artery occlusion, surrounded neuronal loss, and glial scarring (10). In ischemic stroke, the most relevant inflammatory-cellular component is the microglial and astrocytic responses, chemokines and cytokines, and infiltrating peripheral blood cells (9).

There are two types of models in ischemic stroke: the middle cerebral artery (MCA) occlusion model and the photothrombotic MCA stroke model. The photothrombotic MCA stroke model is created by a laser beam irradiating a photosensitizing dye in the MCA. The latter can slowly substitute the former because of the ease of application and reproducibility of the model. However, the latter delays microglial and astrocytic invasion of the ischemic core but elevates the levels of inflammatory cytokines or chemokines and their infiltration from the circulatory system (11). In an experimental striatal stroke model induced by endothelin-1, focal ischemic neuronal loss appeared, with intense microglia activation in 3–14 postlesion days (maximum at 7 postlesion days). Astrocytosis was also maximal at 7 postlesion days (12). In ischemic brains, local inflammation involves astrocytes, activated resident microglia, and infiltrating monocytes or monocyte-derived macrophages (MDMs), with upregulated expression of proinflammatory factors [interleukin (IL)-6, nitric oxide synthase-2, IL-1β, tumor necrosis factor alpha (TNF-α)] and anti-inflammatory factors (CCL22,Ym1, CXCL13,TGFβ,CD163) (13).

#### Astrocytes

Astrocytes are the largest specialized cells in the central nervous system (CNS). Astrocytes play a significant role in neural development and neuroprotection via supporting synaptic connections, ionic homeostasis, and glutamate clearance. It is considered that astrocytes are involved in the local inflammatory response via modulating proinflammatory and anti-inflammatory cytokines (14–16). It was found that astrocytes can enhance neuronal viability through the uptake of glutamate and the release of neurotrophins; astrocytes also compromise neuronal viability by producing inflammatory cytokines or releasing glutamate, and contribute to angiogenesis and neuronal plasticity several days after stroke (17).

The ring- or crescent- shaped “peri-infarct” form is mainly localized around the infarct region and significantly grows after stroke. Microglia and macrophages are mainly localized in the lesion infarct core, rather than in the infarct region (18). One of the pathological alterations of the infarct region is reactive astrogliosis and the formation of glial scarring. Astrocytes in the “peri-infarct” region respond adaptively to stroke, which is known as reactive astrogliosis. Astrocytes can proliferate and be centrally involved in glial scar formation in the “peri-infarct” region, which separates the damaged infarct tissue from the normal tissue. The intertwined connection of astrocytes in and around the infarct region forms the mature glial scar and impedes neuronal rehabilitation after stroke. Early dysfunction and subsequent function recovery after stroke, through the destruction and remodeling of intertwined connection around the infarct region, is associated with neuroinflammation (18, 19).

Reactive astrogliosis and glial scar formation after stroke is considerable during the rehabilitation process, with a change in gene expression, morphology, and proliferation of reactive astrocytes (14, 16). In addition, the main characteristic of astrogliosis is hypertrophic astrocytes with a high expression of proinflammatory cytokines, neurotrophic factors, and neuronal and proliferation markers (8). As major components of the neuroinflammatory process after ischemic stroke, reactive astrocytes have both positive and negative effects on pathological progression (18). Reactive astrogliosis actively protects the neurons in the CNS and regulates their homeostasis to limit the size of the infarct region in the early stage of ischemic stroke. However, if not resolved in time, reactive astrogliosis can also inhibit plasticity and regeneration in the CNS (20). At the early stage of ischemia, perivascular astrocytes can release excess cytokines, which subsequently activate metalloproteases and disrupt the blood-brain barrier (BBB) and vasogenic edema. At the later stage of ischemia, perivascular astrocytes can uptake excess extracellular glutamate, contributing to the regeneration of the BBB (21).

After ischemic stroke, maladapted morphological and functional plasticity of astrocytes occurs in the neurovascular unit, which may result in disorders of the neurovascular unit and disrupt the BBB and astrocyte membrane homeostasis in the CNS during stroke rehabilitation (22). In response to oxidative stress, a typical feature of reactive astrocytes is the high expression of intermediate filament proteins (nanofilament proteins) and remodeling of the intermediate filament system in astrocytes, with a high expression of many characteristic morphological hallmarks. A characteristic morphological hallmark of reactive astrocytes is the presence of hypertrophic astrocytes with increased production of nanofilament proteins, glial fibrillary acidic protein, vimentin, nestin, and synemin (20, 23). Another typical feature of reactive astrocytes is the expression and remodeling of ion channels, which modulate the function of astrocytes by altering the transporters and neurotransmitter receptors. Consequently, alterations in neuronal excitability might lead to secondary neurological disease, such as ischemia and epilepsy during stroke rehabilitation (10). Although astrocytes are not electrically-excitable cells, they can mediate neuron-glia bidirectional interactions through modulating the Ca^2+^ signaling of synapses. It was reported that astrocytes can enhance Ca^2+^ excitability and modulate synaptic function and plasticity during stroke rehabilitation (16). G protein-coupled calcium-sensing receptor expression is also a feature of reactive astrocytes with astrocyte hypertrophy and high expression of glial fibrillary acid protein in ischemic stroke (24).

#### Microglia

Microglia are resident immune cells involved in physiological and pathological processes in the CNS. Physiologically, microglia are long-living resident immune cells that support a stable chemical and physical microenvironment in the CNS. Pathologically, microglia are dynamic immune cells that respond to nervous damage, repair, and regeneration in the CNS. Microglia can be activated and recruited by the injury signals or stimulation and can elicit a quick response to infection or injury by releasing proinflammatory or anti-inflammatory cytokines. The BBB in the CNS consists of microglia, astrocytes, endothelial cells, and pericytes and selectively separates the sensitive brain parenchyma from the circulatory system. Microglia bidirectionally survey the influx of blood-borne components into the CNS and may stimulate the BBB to open, to extravasate leukocytes resulting in angiogenesis (15, 25, 26).

Another pathological change in ischemic stroke is the activation of resident microglia and infiltrating monocytes/macrophages (27). Activated microglia have both positive and negative effects on the pathological progression of ischemia. Early activated microglia contribute to ischemic injury by releasing TNF and IL-1 and can engulf the cellular debris and invading pathogens. Activated microglia also contribute to resolving the inflammatory response by producing IL-10 and TGFβ and inhibiting the ischemia-induced astrocytic response as a neuroprotective effect during stroke rehabilitation (21, 28, 29). Activated microglia participate in attenuating neuronal apoptosis and enhancing neurogenesis after ischemic stroke (30) and they can contribute to nervous reconstruction and repair during stroke rehabilitation together with reactive astrocytes (4). Nevertheless, chronically activated microglia may cause neuronal death by releasing excessive inflammatory mediators (28). Activated microglia appear after ischemia, and microglial survival depends on signaling through the colony-stimulating factor I receptor during stroke rehabilitation. Therefore, depletion of microglia via colony-stimulating factor I receptor inhibitor PLX3397 exacerbates ischemic infarction and augments the production of inflammatory mediators, leukocyte infiltration, and cell death after ischemic stroke (29).

For instance, P2X4 receptors (P2X4Rs) on microglia modulate the inflammatory response to ischemia. In acute ischemia, P2X4R activation leads to microglial activation and proliferation to exacerbate the inflammatory response of ischemia. In chronic ischemia, stimulation of P2X4Rs on microglia leads to release of brain-derived neurotrophic factor to support synaptic plasticity and strengthen behavioral rehabilitation. Therefore, knockout of P2X4R on microglia protects against stroke at the early stage of ischemia but exacerbates behavioral recovery at the late stage of ischemia (27).

Modulating microglial overreaction and microglia-mediated neuroinflammation is considered a therapeutic strategy against ischemic damage. For instance, triggering receptor expressed on myeloid cells 2 (TREM2) was mostly expressed in microglia, but not in neurons, astrocytes, or oligodendrocytes in ischemic stroke. TREM2 responds to inflammation after ischemia to protect against cerebral ischemia/reperfusion. Targeting TREM2 to inhibit the inflammatory response in ischemic stroke may be a new therapeutic option (31). Electroacupuncture is also reported as a safe and effective therapy to attenuate the overactivation of Iba-1 and ED1 positive microglia and the expression of TNF-α, IL-1β, and IL-6 and leads to reduced neurological and sensorimotor impairment in ischemia (32).

#### MDMs

MDMs recruited to the injured area at the early stage of ischemia contribute to behavioral rehabilitation by resolving the inflammatory response. The infiltrating monocytes compromise the neurogenesis from endogenous new striatal neurons from neural stem/progenitor cells. The depletion of circulating monocytes early after ischemic stroke most likely increases the short-term survival of the newly formed neoblasts to enhance neurogenesis, using the anti-CCR2 antibody MC21 (33). Incubation of exogenous peroxiredoxin with murine RAW264.7 macrophages leads to nuclear translocation of transcription factor κB p65 and production of proinflammatory mediators (NO, TNF-α, IL-6) (34). Transcription factor κB is also essential to the upregulation of pro-inflammatory genes, which participate in microglial activation and proliferation during stroke rehabilitation (35).

#### Two Phenotypes of Microglia and MDMs

Microglia and MDMs differentiate toward two phenotypes: the M1 phenotype is the classical one, pro-inflammatory, and detrimental, whereas the M2 phenotype is the alternative one, anti-inflammatory, and protective. The two phenotypes of microglia and MDMs suggest their dual roles. The M1 phenotype, which is activated by toll-like receptors or IF-r, promotes injury, whereas the M2 macrophage or N2 neutrophil phenotype, which is activated by regulatory mediators, such as ILs 4, 10, 13; or TGFβ, prompts tissue remodeling and repair (dualistic role) (21).

These mononuclear phagocytes including microglia and macrophages respond to ischemic stroke dynamically, from the M1 phenotype to the M2 phenotype. After stroke onset, monocytes and microglia infiltrate into the infarct core, peaking 3 days after stroke. Before day 7, MDMs with the pro-inflammatory phenotype dominate, and at day 7, half of the infiltrating MDMs are found to be of the proinflammatory phenotype and the other half of the anti-inflammatory phenotype, but the anti-inflammatory phenotype dominates during the subsequent 2 weeks. Similarly, microglia are predominantly of the proinflammatory phenotype at days 3 and 7 after stroke (12, 36). Therefore, instead of broad suppression, there is a need of shifting the polarization of microglia/macrophages into the protective, anti-inflammatory M2 phenotype during stroke rehabilitation (36, 37). For example, ST2, a member of the IL family, and its ligand IL-33 play critical roles in neuroinflammatory responses after ischemic stroke. There is increased expression of ST2 in microglia during stroke rehabilitation, which enhances the expression of M2 polarization markers on microglia/macrophages and impairs the expression of M1 polarization markers. The absence of ST2 shifted the polarization of microglia/macrophages into a proinflammatory M1-like phenotype. There is also increased expression of IL-33 in astrocytes during stroke rehabilitation, and IL-33 and ST2 serve as immune regulatory brakes on the process of stroke rehabilitation (7).

#### Cytokines

Some cytokines and chemokines have been found to affect the inflammatory response to stroke in the process of stroke rehabilitation. Two important inflammatory mediators of the neuroinflammatory response during stroke rehabilitation are TNF-α and IL-1. We next describe how these two cytokines affect stroke rehabilitation.

##### TNF-α

A common proinflammatory cytokine is TNF-α, which is involved in every phase of the stroke rehabilitation process. When there are certain stimuli, such as ischemia or hemorrhage, TNF-α is synthesized and released by astrocytes, microglia, or neurons in response to the stimuli and is involved in many pathophysiological processes of ischemic stroke or ICH. TNF-α can activate microglia and astrocytes and have a modulatory effect on BBB permeability, and may also have several positive and negative effects on synaptic transmission and synaptic plasticity during stroke rehabilitation (1, 38).

Inhibition of TNF-α R1 signaling can reportedly preserve brain plasticity during stroke rehabilitation. Etanercept, which is a biologic TNF antagonist, can decrease microglial activation in experimental stroke models and has been used therapeutically in animal stroke models. It has been shown that intravenous administration of etanercept is not therapeutic during stroke rehabilitation because biologic TNF inhibitors can be reengineered for BBB penetration. However, intravenous IgG-TNFR fusion protein is reported to have a therapeutic effect on stroke rehabilitation by significantly reducing stroke volume and neural damage (1, 39, 40).

##### Interleukin-1

Another common inflammatory cytokine is IL-1, which affects both systemic and local inflammation and is also an important driver of central and peripheral immune responses to infection or injury. There is considerable experimental and clinical evidence that it is valuable to inhibit IL-1 by IL-1 receptor antagonism as an effective treatment in ischemic stroke. The IL-1 receptor antagonist appears to be a promising treatment target in stroke and is being studied for its therapeutic potential (41).

#### MicroRNAs

MicroRNAs (miRNAs) are small non-coding RNA molecules that regulate gene expression post-transcriptionally by inhibiting the translation of select target genes. MiRNAs are involved in chronic microglial inflammation and lead to progression of neurological diseases such as Alzheimer's disease, amyotrophic lateral sclerosis and stroke (15). In astrocytes and microglia, miRNAs are critical regulators in the mitochondrial response to ischemic stroke. Thereby, MiRNA-targeted therapies have become a viable intervention to optimize mitochondrial function in astrocytes and microglia and improve clinical outcome after ischemic stroke (42).

MiRNA-124 and MiRNA 133b are reportedly involved in the inflammatory response in stroke rehabilitation. Their effects are discussed in detail below.

##### MiRNA-124

MiRNA-124 is the most common brain-specific MiRNA in the CNS and has recently been reported to shift the polarization of activated microglia and infiltration of macrophages into the anti-inflammatory M2 phenotype and while also maintaining microglial activation in the acquiescent state. Early injection of MiR-124 significantly increases the number of microglia/macrophages of the M2-like phenotype and neuronal survival and reduces ischemic core formation by inhibiting reactive astrocytes (36, 37). Moreover, liposomated miR-124 administration before the peak of the proinflammatory process in ischemic stroke can shift the predominantly proinflammatory microglia/macrophage phenotype into the anti-inflammatory M2 phenotype most effectively and enhance stroke rehabilitation in the subacute phase (36).

##### MicroRNA 133b

Compared with naïve multipotent mesenchymal stromal cells (MSCs), MSCs with overexpressed MiRNA 133b significantly contribute to stroke rehabilitation in animal models of MCA occlusion. Exosomes releasenaïvenaive MSCs are beneficial mediators in the MSC treatment of ischemic stroke. Ex-miR-133b+ significantly increases the release of exosomes from astrocytes by promoting neurite branching and elongation of cortical embryonic neurons, whereas Ex-miR-133b- significantly decreases the release (43).

### ICH

ICH is the most critical subtype of stroke and lacks effective treatment (44). ICH also leads to neuronal loss, cerebral edema, and neuropathological alterations, including activation of astrocytes and microglia/macrophages and the invasion of neutrophils and T lymphocytes from the blood circulation after ICH (34). There are two types of ICH models: the collagenase-induced model and the autologous arterial whole blood-induced model (45). ICH also leads to neuronal loss, cerebral edema, and neuropathological alterations, including microglial/macrophage and astrocytic activation, and neutrophil and T lymphocyte invasion after ICH (34). After ICH, microglia and astrocytes in brain tissue adjacent to the hematoma may modulate brain cellular plasticity (3). Microglia are among the first non-neuronal cells in the innate immune response to ICH. Microglia become activated by the classical pro-inflammatory M1 phenotype or alternative anti-inflammatory M2 phenotype (44). Astrocytes have differential roles in the recovery pattern of ischemic and hemorrhagic stroke. However, there is similar long-term GFAP-positive astrocytic plasticity after both ischemic stroke and ICH (46). Astrocyte HO-1 overexpression shows distinct neuroprotection after ICH (47). Moreover, ICH stimulates expression and release of Prx 1, activation of toll-like receptor4/nuclear factor κB, and production of cytokines (TNF-α, IL-6, and IL-17) (34). Prostaglandins such as PGE2 also mediate secondary brain injury in the inflammatory response to ICH. The EP2 receptor, which can be activated by PGE2, is expressed in neurons but not in astrocytes or microglia after ICH. The neuronal EP2 receptor shows neuroprotection after ICH by suppressing inflammatory responses, oxidative stress, and matrix metalloproteinase-219 activity, which is involved in brain injury after ICH (45).

## PSD

PSD is a critical psychiatric complication after stroke that frequently occurs at ~3–6 months and remains for 2–3 years after ischemic stroke or ICH. It is reported that the prevalence rate of PSD is ~33% in ischemic stroke and it is 15% at 1 year after ICH. It is independently associated with increased morbidity and mortality in stroke because PSD may hinder rehabilitation. To wit, alleviating PSD can improve the outcomes and quality of life in patients after stroke. PSD is reportedly associated with late worsening of disability, but not with initial damage severity after stroke (4, 48, 49).

The mechanisms between cerebrovascular diseases and depressive disorders are intertwined. As the inflammatory response in stroke affects stroke rehabilitation, some studies have confirmed that an immunological hypothesis is one of the pathophysiological mechanisms of PSD and the inflammatory response in PSD affects its outcome. However, the specific mechanisms of the inflammatory effects on stroke and PSD reportedly differ. It has been shown that patients with PSD have early increased inflammatory markers (such as high-sensitivity C-reactive protein, ferritin, neopterin, and glutamate), increased proinflammatory cytokines (TNF-α, IL-6, IFN-γ), increased pro-inflammatory/anti-inflammatory ratios (TNF-α/IL-10, IL-6/IL-10), and lowered complement expression (5). Recent investigations have revealed imbalances in inflammatory cytokine levels and increased oxidative DNA damage in association with PSD, as well as the involvement of inflammatory and immune mechanisms and elevated oxidative stress level in PSD (49–52). It has also been reported that cytokines can drive intrinsic apoptotic factors to increase the risk of PSD through intracellular calcium and glutamate excitotoxicity after ischemic damage; pro-inflammatory cytokines may amplify the proinflammatory processes by activating indoleamine 2, 3-dioxygenase and reducing serotonin production, which sequentially results in PSD. Cytokines can provoke the dysregulation of several growth factors, such as BDNF, fibroblast growth factor-2, and contribute to PSD and other comorbidities (53–55).

## Pharmacotherapy

### Pharmacotherapy in Stroke Rehabilitation

Using anti-inflammatory drugs along with neurorehabilitation therapy is useful for neuroprotection and functional recovery (56). Anti-inflammatory drugs may enhance brain plasticity after stroke but need to be used in conjunction with neurorehabilitation therapy (57). Therefore, anti-inflammatory treatment has the most potential as a therapy to enhance neurorehabilitation after stroke (58).

The findings of recent studies on anti-inflammatory treatments for stroke are listed below. Multimodal intervention of minocycline (pharmacotherapy, anti-inflammatory drug used to modulate the dynamics of the immune system) together with cerebral stimulation using transcranial direct-current stimulation and repetitive transcranial magnetic stimulation (neurorehabilitation therapy used to enhance functional recovery after stroke) may augment plasticity, rehabilitation, and neurorestoration (48). Simvastatin (statin class of cholesterol-lowering drugs), alters the release of cytokines and trophic factors from microglia, including IL-β, TNF-α, and brain derived neurotrophic factor in a cholesterol-dependent manner, but inhibits phagocytosis in a cholesterol-independent manner (59). Vinpocetine (a potent anti-inflammatory agent) improves neuronal plasticity and reduces the release of inflammatory cytokines and chemokines from microglia, macrophages, endothelial cells, and vascular smooth muscle cells (60). Omega-3 polyunsaturated fatty acids provide anti-inflammatory neuroprotective function in ischemic stroke by targeting astroglia and microglia and preventing the release of cyclooxygenase 2, hypoxia-inducible factor 1α, nitric oxide synthase, and IL-1β and have clinical potential as a therapeutic treatment in stroke (61). Trypsin inhibitor ulinastatin (anti-inflammatory drug) provides neuroprotective function in synaptic plasticity and spatial memory in cerebral ischemia-reperfusion injury (62). The melanocortin MC4 receptor agonist RO27-3225 (used to reduce expression of TNF-α, BAX, ERK1/2, JNK1/2, and cascapse-3 and counteract prolonged/recurrent inflammatory and apoptotic responses) provides neuroprotective function and promotes functional recovery in ischemic stroke (63). Scutellarin (a potential Chinese herbal extract, a putative therapeutic agent, used to improve neurological function), ameliorates neuroinflammation by suppressing microglial activation and enhances astrocytic reaction by upregulating the expression of neurotrophic factors (8, 28).

Sex differences are involved in the frequency of intracellular astrocyte Ca2+ elevation and microglial volume immediately in ischemic stroke and are foundational for future sex-specific stroke therapeutic treatments (64). Female sexual hormones (estradiol and progesterone, anti-inflammatory), modulate the cellular and immune response to ischemic stroke (9). The corticotropin-releasing hormone type 1 receptor actively alters neuronal injury and inflammation, neuronal plasticity, and functional recovery in ischemic stroke (65).

The vascular endothelial growth factor mediates reactive astrocyte transdifferentiation into new mature neurons and enhances neurogenesis in ischemic stroke (66). The vascular endothelial growth factor also suppresses the inflammatory response in ischemic stroke to promote neuronal plasticity and neuronal remodeling (67). High-mobility group box 1 (amphoterin or HMG1) promotes neuronal necrosis and influx of damaging inflammatory cells in the acute stage of ischemic stroke but promotes beneficial plasticity and neuronal recovery in the delayed stage after stroke (68). The vagus nerve system regulates the immune system through the cholinergic anti-inflammatory pathway (69). Acetylcholine-alpha 7 nicotinic acetylcholine receptor on macrophages or microglia also provides neuroprotection through the cholinergic anti-inflammatory pathway. Nicotine (an acetylcholine receptor agonist, anti-inflammatory), inhibits microglial activation and production of proinflammatory cytokines and cholinesterase by activated astrocytes, which is partly medicated by COX-2 (70). Therefore, treatments inhibiting cyclooxygenases enhance neuronal plasticity after ischemic stroke (71). Therapeutic hypothermia is another potential treatment for ischemic stroke. Novel neurotensin receptor1 (NTR1) agonists induce hypothermia to inhibit microglial activation and decrease the expression of proinflammatory (M1) chemokines and cytokines and protect against neuronal damage in ischemic stroke and ICH (6).

### Pharmacotherapy in PSD

Anti-cytokine modulators are new therapeutic targets for the treatment of PSD, especially in subjects affected by inflammatory processes. For instance, an investigation revealed that anti-inflammatory treatment, such as acetylsalicylic acid, non-steroidal anti-inflammatory drugs, and statins decrease the risk of PSD, and inflammation contributes to PSD depending on the onset of PSD (72).

## Conclusions

Stroke comprises ischemic stroke and ICH. The immuno-inflammatory process is involved in neural plasticity following events such as a hemorrhage or ischemic stroke. After ischemia, astrocytes, microglia, and MDMs play important roles during rehabilitation with the modulation of cytokines or chemokines, such as TNF-α and IL-1. Moreover, MiRNAs are also important posttranscriptional regulators in these glial mitochondrial responses to cerebral ischemia. ICH involves processes similar and different to those seen in ischemia, including neuronal injury, astrocytic and microglial/macrophage activation, and neutrophil and T lymphocyte invasion after ICH. Immunological hypothesis is also one of the pathophysiological mechanisms of PSD. To date, many pharmacotherapies have been suggested as having an anti-inflammatory function in stroke rehabilitation, including those involving minocycline, melanocortin, omega-3 polyunsaturated fatty acids, UTI, statin, vinpocetine, RO27-3225, scutellarin, and sexual hormones. Other potential therapies involve the vascular endothelial growth factor, high-mobility group box 1, corticotropin-releasing hormone type 1 receptor, the vagus nerve system, nicotine and cyclooxygenase 2, and therapeutic hypothermia. In PSD, very few anti-inflammatory treatments have been studied, including the use of acetylsalicylic acid, non-steroidal anti-inflammatory drugs, and statins.

## Author Contributions

MF, LZ, and XJ wrote the first draft. RC and WY provided the organization and framework of the article. SG, JL, BL, and TL provided the critical revisions. All authors approved the final version of the manuscript for submission.

### Conflict of Interest Statement

The authors declare that the research was conducted in the absence of any commercial or financial relationships that could be construed as a potential conflict of interest.

## Abbreviations

BBB: blood-brain barrier
CNS: central nervous system
ICH: intracerebral hemorrhage
IL: interleukin
MCA: middle cerebral artery
MDM: monocyte-derived macrophage
MiRNA: microRNA
MSC: multipotent mesenchymal stromal cell
PSD: poststroke depression
TNF-a: tumor necrosis factor alpha.
